# Health-Related Hot Topic Detection in Online Communities Using Text Clustering

**DOI:** 10.1371/journal.pone.0056221

**Published:** 2013-02-15

**Authors:** Yingjie Lu, Pengzhu Zhang, Jingfang Liu, Jia Li, Shasha Deng

**Affiliations:** 1 Antai College of Economics and Management, Shanghai Jiao Tong University, Shanghai, China; 2 School of Business, East China University of Science and Technology, Shanghai, China; The University of Auckland, New Zealand

## Abstract

Recently, health-related social media services, especially online health communities, have rapidly emerged. Patients with various health conditions participate in online health communities to share their experiences and exchange healthcare knowledge. Exploring hot topics in online health communities helps us better understand patients’ needs and interest in health-related knowledge. However, the statistical topic analysis employed in previous studies is becoming impractical for processing the rapidly increasing amount of online data. Automatic topic detection based on document clustering is an alternative approach for extracting health-related hot topics in online communities. In addition to the keyword-based features used in traditional text clustering, we integrate medical domain-specific features to represent the messages posted in online health communities. Three disease discussion boards, including boards devoted to lung cancer, breast cancer and diabetes, from an online health community are used to test the effectiveness of topic detection. Experiment results demonstrate that health-related hot topics primarily include symptoms, examinations, drugs, procedures and complications. Further analysis reveals that there also exist some significant differences among the hot topics discussed on different types of disease discussion boards.

## Introduction

Research about health communication has demonstrated that patients are increasingly using the Internet for health information and support. A study of US-based cancer patients and their caregivers indicated that 80% of them were interested in health-related information on the Internet and 65% expressed an interest in online support groups [1]. Especially in recent years, with the advent of social media services such as Wikipedia, Facebook, online forums and message boards, patients are more likely to obtain health information and share health experiences on these social media websites [2]. A recent survey [3] demonstrated that 80% of Internet users have searched online for information about health topics, such as a specific disease or treatment, 34% of them have read someone else’s commentary or experience about health or medical issues in online news group, website, or blog, and 24% of them have consulted online reviews of particular drugs or medical treatments.

There are multiple reasons why patients and their caregivers use the Internet, especially social media services, for health information. (1) Patients feel that doctors are too busy to answer their questions [4], and many doctors tell their patients basic medical information but are not willing to take the time to fully explain the details [5]; this view is supported by the argument of Tyson [6], who suggests that there is a lack of attention to detail in the current doctor-patient relationship. (2) The Internet enables patients to take a more active role in making decisions about their health through the use of social support and the ability to explore treatment options [7]. Patients with chronic diseases are especially likely to search for online health information to be better informed about their illnesses [8]. (3) Convenience and anonymity [4] are important reasons why patients use the Internet. Patients expect to obtain health-related knowledge easily and quickly, and they are not embarrassed to ask health professionals online or communicate with online members about their conditions [9].

Although different types of social media applications can be used to obtain health-related information, online health communities are among the most popular social media services. In online health communities, patients and their caregivers can share their experiences and exchange interesting information. The emotional support and encouragement offered by community members is also important for patients suffering serious illness and helps them cope with their diseases significantly better than those who address serious diseases by themselves.

A thorough understanding of the interests, motivations and behaviors of these online health consumers could be important for many domains. For the websites that provide health-related social media services, a better understanding of how people participate in the online discussions could assist the web designers and developers in optimizing the human-computer interface, providing personalized tools and functions to facilitate patient engagement and improving the ease of use and social interaction. Characterizing patients’ online behaviors could assist information analysts and researchers in clearly summarizing the present situation, revealing existing problems, and planning the developmental direction of online health communities. More importantly, the study is of great help to the end users of online health communities themselves, especially the newcomers. Newcomers might find it difficult to immediately understand this new form of online communication, so health topic analysis enables them to obtain a sense of what online health communities are, quickly find the issues they concerned about, and become involve in online health communities more easily, thereby gaining valuable information for their health self-management.

For these reasons, there are many studies on determining hot topics in online health communities using different research methods, such as survey methods based on questionnaires and statistical content analysis. In earlier studies, categories or themes of information shared in Internet medical support groups were determined according to the number of people who used the list and how frequently they posted on it [10]. Later, many surveys were developed to evaluate the use of web-based medical information resources by different user groups. Some adopted the methods of experimental study, questionnaires and interviews to statistically analyze interesting topics [11]. However, survey research is always based on a sample of the population, and survey samples are usually self-selected; thus, the population characteristics often cannot be inferred accurately. With the rapid development of some healthcare websites, some case studies characterized the health-related messages on targeted websites by extracting the online messages posted by community members and analyzing the prevalence of different types of medical information. Most studies focused on chronic diseases such as Parkinson’s disease and common high-mortality diseases such as breast cancer [12]–[13]. Previous studies demonstrated that the most frequent themes with which patients are concerned are prevention and diagnosis, treatment, support and long-term side effects of treatment. However, this statistical content analysis is based on manual annotation, which requires significant human effort and is thus labor-intensive, expensive, time-consuming and often error-prone. Therefore, when faced with the tremendous amount of health-related information available in online communities, it is becoming difficult to employ traditional statistical approaches to explore health-related topics [14].

Recently, some topic analysis techniques have been widely used to process medical text. Previous topic exploration based on text mining of medical text primarily focused on clinical narratives and medical literature. To find the interesting medical literature from the MEDLINE biomedical literature database, Lin [15] designed an automatic document clustering method to divide the retrieved literature into different topical groups and prioritized the important literature in each group. To help diabetic patients find appropriate patient educational materials, Kandula [16] presented a method for matching patient education materials to patient clinic notes through topic modeling such that relevant education articles could be recommended automatically to the patients. The study by Patterson [17] demonstrated that document clustering is a feasible method to clearly differentiate the types of clinical narratives. The studies above primarily focused on professionally written medical text. Document clustering, as used for this type of medical text, aims to provide a tailored presentation of the relevant medical text and facilitate the users’ searches. In recent years, however, user-generated medical text has been shared on many social media services, including medical weblogs, health Q&A, and online health communities. Some studies applied text-mining techniques to this user-generated medical text to explore the topics that interest online health information consumers. Denecke [18] focused on medical weblogs and classified the topics in medical weblogs into two types: informative and affective. Brody [19] used text classification based on Latent Dirichlet Allocation(LDA) topic models to detect the salient aspects of online reviews of health professionals. Chen [20] performed a cluster analysis on medical posts from three online health communities and found that the clusters could be classified into a set of common categories: generic, support, patient-centered, experiential knowledge, treatments or procedures, medications and condition management. In his study, the clustering method proved useful for identifying different types of topic information. However, this user-generated medical text from social media sites differs significantly from professionally written text. General document clustering techniques thus do not produce satisfactory results when distinguishing health-related topics because of the users’ lack of medical knowledge.

To address these gaps, we propose a novel approach for health-related hot-topic detection using text clustering. Our aim is to automatically distinguish different health-related topics in online health communities more effectively. Meanwhile, we hope to determine the differences in interesting topics among different types of disease discussion boards using our text clustering method.

## Methods

### Test Site and Sample

In this study, we used Medhelp.org as our data source. Medhelp.org is one of the most popular online health communities. It consists of over 230 discussion boards that concern different diseases. Community members are composed primarily of patients and their caregivers, who seek health-related information, and some individual health professionals, who share their experiences and knowledge. A small number of doctors also offer their expertise and answer questions from community members. Since the site’s creation in 1994, nearly 3 million threads have been posted in the community, and the site attracts over 12 million visitors every month. The site has also been selected as an experimental data source in previous studies [21].

Next, some representative discussion boards must be determined. Lung cancer and breast cancer are the most common cancers with high mortality, and some studies demonstrate that both cancers are among the most common cancers about which Internet users search for information [22]. Diabetes, one of the most common chronic diseases, is also among the most frequently discussed diseases in online healthcare communities. Thus, this paper chose lung cancer, breast cancer and diabetes as research subjects and collects the messages posted in these three disease discussion boards (Table 1). To evaluate our approach, we manually annotated the messages and classify them into different semantic categories based on their health-related topics.

**Table 1:** Data collection statistics.

Disease type	Messages	Members	Messages per member	Time span
Lung cancer	4,728	1,928	2.45	March 2004–March 2012
Breast cancer	65,856	16,100	4.09	March 2004–March 2012
Diabetes	25,509	8,169	3.12	March 2004–March 2012

**Figure 1:**
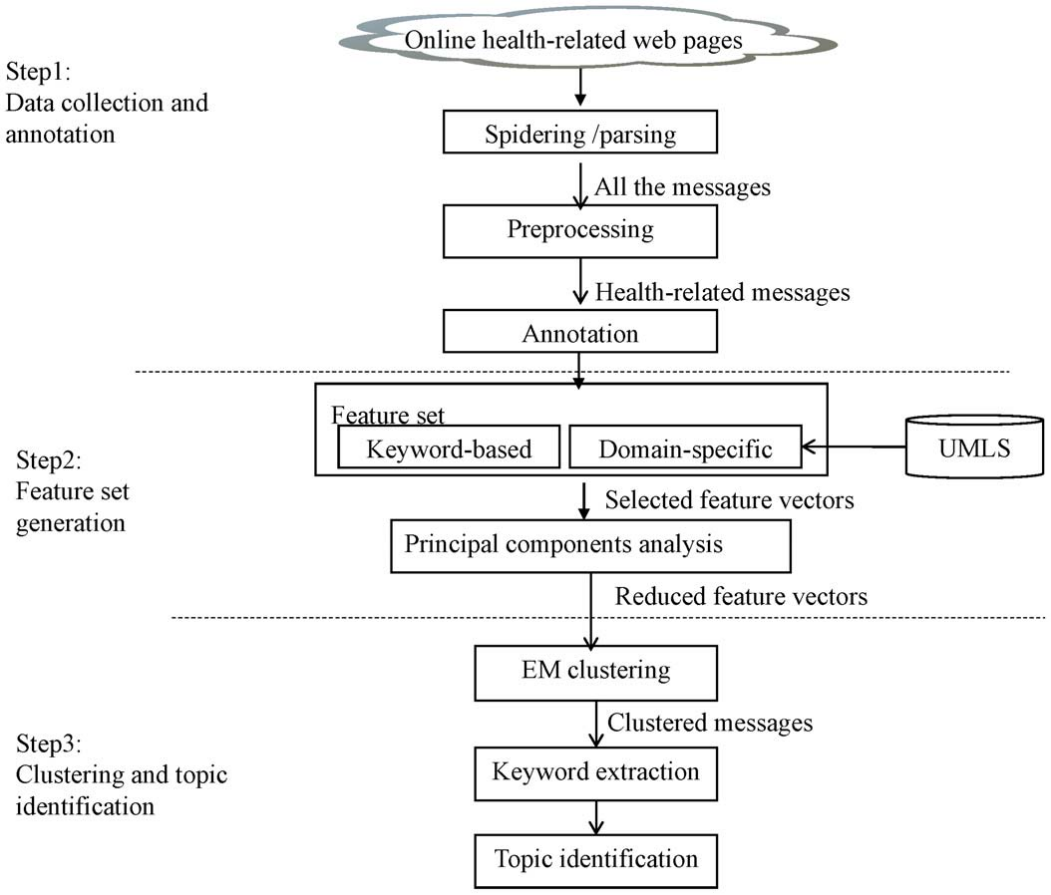
The design of the topic analysis method.

We proposed a method to automatically determine health-related hot topics in online health communities. Our research method consisted of three steps: data collection and annotation, feature set generation, and clustering and topic identification, as shown in Figure 1.

### Data Collection and Annotation

In the data collection step, we used the web crawler software Offline Explorer to obtain all the web pages from the discussion boards. Then, we parsed the pages to extract available messages and stored the messages into a database. Next, some noisy and unreliable data were filtered by text preprocessing, including stop words removal and word stemming. Furthermore, to produce better topic clustering results, some messages unrelated to health topics were filtered. For example, many patients posted messages to seek sympathetic encouragement, share positive thoughts, and show compassion or empathy for their peers, such as “*Thank you for your answer*” or “*I hope you get well soon*”. These messages of emotional expression contain no medical information and should be removed.

After preprocessing, all messages were independently annotated by two annotators which were professional and experienced health experts. These messages were classified into different topic groups according to the pre-specified categories. And finally Cohen’s Kappa was calculated to check for the inter-agreement between the two annotators.

### Feature Set Generation

Document clustering has been widely used to explore medical topics in biomedical informatics. The vector space model (VSM) [23] was considered to be an effective method for modeling text content. In the VSM, text is represented by a vector of terms, which are typically keywords and phrases. Therefore, we also used these keywords as part of the text features. Additionally, because health-related messages posted in online communities contained much medical knowledge, incorporating medical domain-specific text features as additional features could significantly enhance the clustering performance. Previous studies have often used the UMLS Metathesaurus, the world’s largest repository of biomedical concepts, to extract medical terminology. It consists of 1.7 million biomedical concepts, where each concept is assigned to at least one of the 134 semantic types. To avoid an extraction of very general concepts, we further analyzed the health-related terms in the messages and their semantic types based on previous studies [18], and extracted the most frequent health-related semantic types as domain-specific features, as listed in Table 2. To score these semantic features, we must extract health-related terminology from the messages and compute the word frequencies of the terminology that belongs to the same semantic type. This study used MetaMap, a highly configurable program that maps biomedical text to concepts in the UMLS Metathesaurus, to obtain these medical terminologies automatically. All the features adopted in our study are shown in Table 3.

**Table 2:** The UMLS semantic types used.

Abbr.	Semantic Types	Abbr.	Semantic Types
aapp	Amino Acid, Peptide, or Protein	lbpr	Laboratory Procedure
acab	Acquired Abnormality	imft	Immunologic Factor
anab	Anatomical Abnormality	inpo	Injury or Poisoning
bdsy	Body System	mobd	Mental or Behavioral Dysfunction
blor	Body Location or Region	neop	Neoplastic Process
bmod	Biomedical Occupation or Discipline	orch	Organic Chemical
bpoc	Body Part, Organ, or Organ Component	patf	Pathologic Function
diap	Diagnostic Procedure	phsu	Pharmacologic Substance
dsyn	Disease or Syndrome	sosy	Sign or Symptom
horm	Hormone	topp	Therapeutic or Preventive Procedure

**Table 3:** Features adopted in the method.

Feature category	Features
Keyword-based	Frequency of unigram words
	Frequency of bigram words
	Frequency of trigram words
Medical domain-specific	Frequency of medical terms
	Frequency of the semantic types

The features extracted from the messages were quantified as feature vectors to be used as input for the topic clustering. However, these vectors were characterized by high dimensionality, redundancy and high correlation among individual attributes, which was unfavorable for clustering [24]. A typical feature reduction technique in text mining, principal component analysis (PCA), can be used to address the problem. PCA assumes that the variance in all attributes is caused by a few core factors, i.e., the principal components. PCA estimates these principal components by calculating linear combinations of the attributes that have the largest variances. The original attributes were replaced with the principal components to reduce the high dimensionality. Interdependency between attributes were also reduced because principal components are orthogonal.

### Clustering and Topic Identification

Many clustering methods have been used in previous studies. In this study, we used expectation maximization (EM) clustering, a type of probabilistic clustering approach that assigns each instance a probability of belonging to each cluster. The EM algorithm was first introduced by Hartley et al. in 1958 [25] and developed by Dempster et al. in 1977 [26]. The EM algorithm generates the first model, and iterative refinement of the data set is performed until the maximum likelihood, i.e., the optimal model, is attained. The probability that an object belongs to a mixture model can be iteratively calculated to determine the optimal model, and the adequateness of the model can be determined using log-likelihood functions. Thus, the EM algorithm is an algorithm for probability-based clustering. Using a parameter 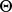, a random variable X of observation results, and a random variable Z that cannot be observed, the probability distribution of (X,Z) can be written as 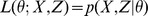. Thus, the following likelihood function must be maximized:
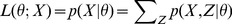
(1)


The iterative approach of the EM algorithm requires the parameter 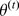 and determines the next-step parameter 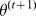. The steps are classified into Expectation (E) and Maximization (M) steps. In the expectation step, the algorithm defines the expectation *Q* of the likelihood function given 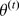.
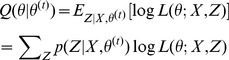
(2)


In the maximization stage, the algorithm calculates 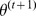. by maximizing *Q*.
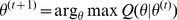
(3)


In the practice, we initialize 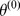 with any proper values (or vectors) and iteratively calculate 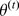 until the solution converges to within an error tolerance.

Most clustering algorithms require the expected number of clusters to be specified in advance, which is problematic because clustering is intended to be an unsupervised learning method; in contrast, EM clustering can evaluate various numbers of clusters and determine the optimal number of clusters by performing cross validation on different numbers of clusters.

To better distinguish different clusters and understand the topics represented by the clusters, some key phrases were selected for topic identification. Key phrases contained medical terminology extracted using the UMLS lexicon and some n-gram (uni-gram, bi-gram and tri-gram) words that appear with high frequencies. A key phrase extraction approach similar to the TF-IDF scheme was used. Let the clusters be denoted C_1_, C_2_, …, C_N_. For each n-gram term w in a cluster C_i_, its score is calculated as
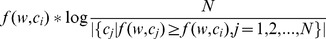
(4)where 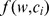 is the frequency of w in cluster 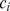, and 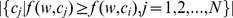 is the total number of clusters with a term w frequency greater than or equal to the term w frequency of the cluster being evaluated.

Once key phrases with high scores were ranked and combined with expert opinions, the topics represented by clustered messages can be identified.

### Evaluation Metrics

An evaluation approach that references external criteria was used in this study to evaluate the results of the clustering [27]. Based on a pairwise comparison with the pre-specified category of the data set, three commonly used metrics, the Rand index, Jaccard coefficient and FM (Fowlkes and Mallows) index, were utilized for performance evaluation. Let SS be the number of pairs of items belonging to the same cluster and category, SD be the number of pairs belonging to the same cluster but different categories, DS be the number of pairs belonging to different clusters but the same category, and DD be the number of pairs belonging to different categories and clusters. SS and DD are “good choices”, and DS and SD are “bad choices”. The three metrics are defined as follows:
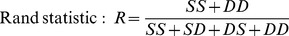
(5)

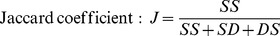
(6)

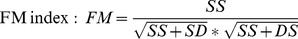
(7)


## Results

The EM clustering algorithm in our study was performed using the Weka software package, a popular suite of machine learning software written in Java and developed at the University of Waikato. After preprocessing and principal components analysis, EM clustering was performed. The Weka implementation of EM provides an option to automatically determine the best number of clusters using 10-fold cross validation. First, the initial number of clusters was set to 1, and the data were divided into ten folds. Next, we calculated the average log-likelihood by executing the EM algorithm independently on every fold. As the number of clusters increased, the process was repeated until the average log-likelihood stopped increasing.

**Figure 2:**
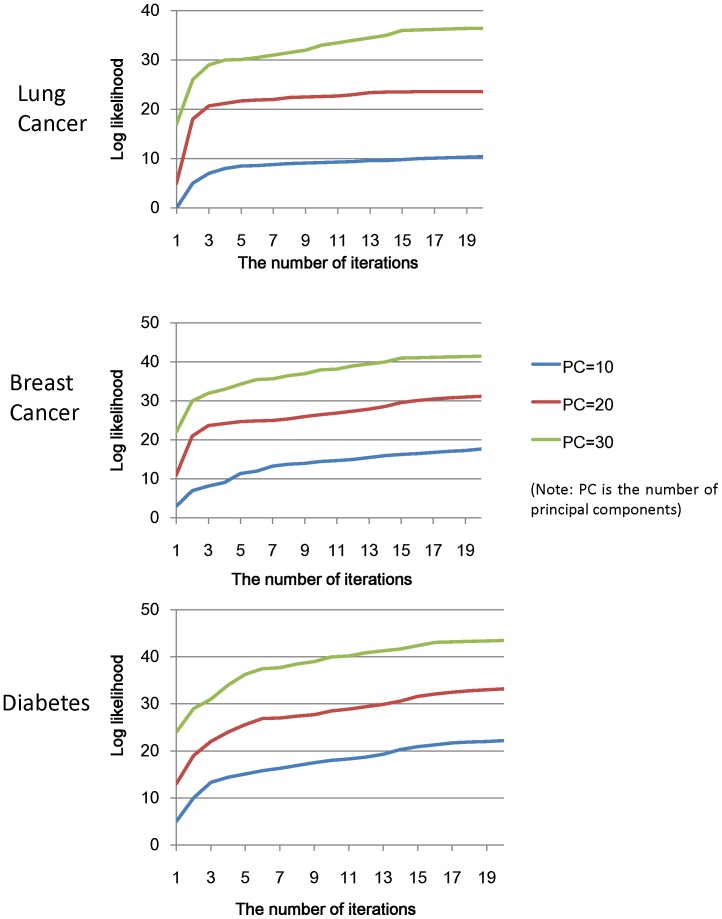
Log-likelihood versus iteration number for the clustering of lung cancer, diabetes and breast cancer.

We also verified that EM clustering progressively determined a better fit to the data by checking the log-likelihood at each iteration. As can be observed from Figure 2, when performing the EM algorithm on the experimental data from the three disease discussion boards, the value of the likelihood increased after each iteration until the EM clustering reached convergence.

After incorporating the clusters with similar semantic types, the main topic groups were identified and ranked for the three disease discussion boards, as shown in Tables 4, 5, and 6. The names of the topic groups were determined according to the extracted key phrases. All five clusters corresponded to some specific UMLS semantic types. The clusters represented by the semantic type sosy (Sign or Symptom) were assigned as to the symptom category. The clusters represented by the semantic types dsyn (Disease or Syndrome) and patf (Pathologic Function) were assigned to the complication category. The clusters represented by the semantic types diap (Diagnostic Procedure) and lbpr (Laboratory Procedure) were assigned to the examination category. The clusters represented by the semantic type topp (Therapeutic or Preventive Procedure) were assigned to the procedure category. The clusters represented by semantic type phsu (Pharmacologic Substance) were labeled as drug.

**Table 4:** Key phrases extracted from lung cancer discussion boards.

Cluster	Label	Key phrases	UMLS semantic types
1	Symptom	pain, symptoms, cough, breathless, chest pain, painful, shortness of breath,coughing up blood, short of breath, wheezing, nausea	sosy
2	Complication	pneumonia, infection, tuberculosis, bronchitis, asthma, COPD, pleural effusion,emphysema, atelectasis, collapsed lung	dsyn, patf
3	Examination	cat scan, biopsy, X-ray, pet scan, chest X-ray, scans, MRI, bronchoscopy,imaging, biopsy needle	diap
4	Procedure	chemo, radiation, chemotherapy, lobectomy, operation, therapy,surgery, removal, radiation therapy, wedge resection	topp
5	Drug	silicas, tarceva, morphine, chantix, carboplatin, coumadin, alimta, advil, taxol, dilaudid	phsu

**Table 5:** Key phrases extracted from breast cancer discussion boards.

Cluster	Label	Key phrases	UMLS semantic types
1	Examination	biopsy, mammogram, ultrasound, MRI, BI-RADS, biopsy needle, core biopsy,cat scan, imaging, screening	diap, lbpr
2	Procedure	chemo, radiation, mastectomy, lumpectomy, chemotherapy,implant, removal, operation, radiotherapy, surgical	topp
3	Symptom	pain, painful, sore, nipple discharge, breast pain, itching, itchy,tingling, hot flashes, nausea	sosy
4	Drug	tamoxifen, arimidex, femara, taxol, taxotere, effexor, carboplatin,raloxifene, valium, docetaxel	phsu
5	Complication	infection, lymph edema, rash, fibrocystic breast, mastitis, IDC, eczema, complex cyst,complex cysts, paget’s disease, neuropathy, fibrocystic disease, fibrocystic breastdisease	dsyn

**Table 6:** Key phrases extracted from diabetes discussion boards.

Cluster	Label	Key phrases	UMLS semantic types
1	Drug	insulin, lantus, metformin, januvia, glucophage, actos, marihuana, avandia, glipizide, amaryl	phsu
2	Complication	hypoglycaemia, low blood sugar, infection, DKA, PCOS, BGs, coma, kidney disease, obesity, diabetic neuropathy	dsyn, patf
3	Symptom	pain, tired, thirsty, nausea, fatigue, tingling, frequent urination, hungry, sore, dizzy, itchy	sosy
4	Examination	blood test, fasting test, glucose test, fasting blood sugar, hemoglobin A1c test, glucose tolerance test, cat scan, GTTS, MRI	lbpr, diap
5	Procedure	infusion, injection, transplant, therapy, dialysis, CDE, RX, amputation, insulin injection, ect	topp

To examine the effects of our clustering method, we further evaluated the results of the clustering based on different feature sets. From Table 7, we can see that clustering using a combination of keyword-based features and medical domain-specific features has outperformed clustering using only keyword-based features significantly, indicating that incorporating the medical domain-specific features as additional features enhanced the performance of clustering significantly.

**Table 7:** Performance measures using different feature sets.

Disease	Feature Set	Rand	Jaccard	FM
Lung Cancer	F1	0.762	0.284	0.460
	F1+F2	**0.844**	**0.394**	**0.570**
Breast Cancer	F1	0.741	0.220	0.361
	F1+F2	**0.825**	**0.359**	**0.530**
Diabetes	F1	0.752	0.246	0.395
	F1+F2	**0.837**	**0.381**	**0.554**

(Note: F1 are keyword-based features and F2 are medical domain-specific features).

## Analysis and Discussion

**Figure 3:**
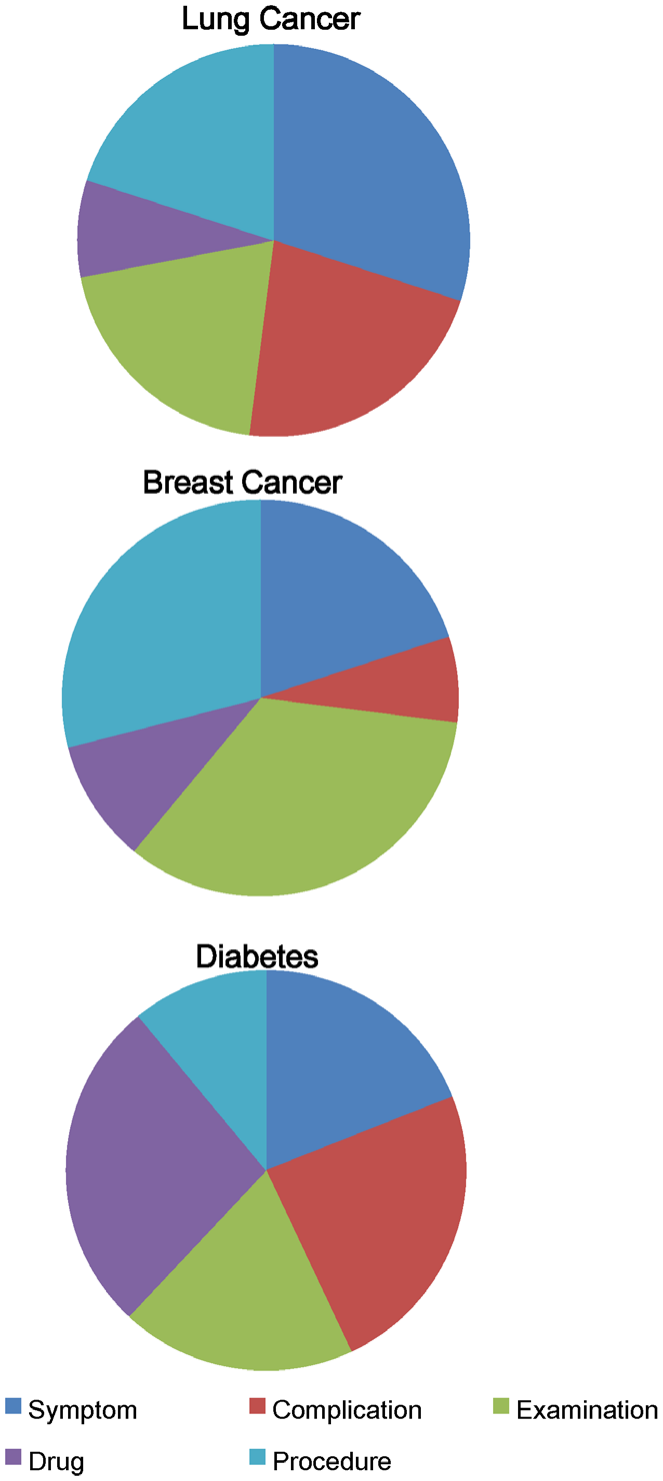
The distribution of hot topics for the discussion boards of lung cancer, breast cancer and diabetes.

To reveal the relationships among the disease types and the five informative topics, we used pie charts to visualize the results. As can be observed from Figure 3, the percentage figures indicated that patients with different types of diseases were interested in different health topics.

The symptom and examination topics are both hot topics related to disease diagnosis. Members of breast cancer discussion boards are more likely to discuss examinations, such as biopsies and mammograms, whereas members of lung cancer discussion boards prefer to discuss symptoms, such as chest pain and coughing. One possible explanation is that breast tissue is on the body’s surface, so people can find abnormalities easily. However, there are no obvious other symptoms in the early stage of breast cancer that support the diagnosis. Thus, those who suspect they have breast cancer are more likely to visit the hospital for clinical examinations. Unlike breast cancer, lung cancer has some early symptoms, such as coughing or wheezing, that are similar to those of other ailments, such as fever and bronchitis. Potential lung cancer patients do not often consider these symptoms as signs of cancer, which makes it unlikely for them to visit the hospital for further examination. Instead, they refer to online communities to consult others about their symptoms.

The distributions of drug and procedure topics are clearly distinguishable for the three disease discussion boards. The drug topic accounts for a significantly higher fraction of the topics discussed on the diabetes discussion board then on the two cancer discussion boards, whereas the procedure topic constitutes a significantly lower fraction of the topics discussed on the diabetes discussion board than on the two cancer discussion boards. This occurs because both cancers are diseases with high mortality and are primarily treated with chemotherapy, radiation therapy and surgery. The procedure topic, therefore, is a major concern for cancer patients; in contrast, drugs are supplementary treatments for cancer patients, so the drug topics constitute a relatively small fraction of the topics discussed. In contrast, diabetes is a common chronic disease, and drug treatment is a main treatment for diabetes. The patients who are long-term users of anti-diabetic drugs prefer to discuss the efficacy and side effects of these drugs. Thus, the drug topic constitutes a large fraction of the topics discussed on the diabetes discussion boards.

The complication topic is another hot topic in online communities. The distribution of complication topics helps us understand that, compared with lung cancer and diabetes patients, breast cancer patients have fewer and less severe complications.

### Conclusions and Future Research

Because of the recent development of online health communities, a thorough understanding of health-related topics could be important for website providers, information researchers and end users. In this paper, we have proposed a method based on clustering analysis technique to explore health-related hot topics in online health communities automatically instead of using the statistical analysis techniques employed in most previous studies. By integrating medical domain-specific knowledge, we have constructed a medical topic analysis model; a case study is used to validate the proposed method as an effective approach for identifying different health-related topics automatically. Several valuable conclusions can be drawn from the experimental results, including that significant differences exist among the topics discussed most frequently on different types of disease discussion boards.

This paper also has some limitations that must be considered. First, the study has demonstrated that using additional medical domain-specific features can enhance the performance of topic analysis significantly; however, other features could be used to further improve the clustering performance. For example, messages within a single thread most likely contain the same topics, so these structural features should be considered in the feature set in the further research. Second, feature sets contain many features, and the irrelevant features can greatly impact clustering performance. However, traditional feature selection algorithms work only for supervised data for which class information is available. We would require significantly more research about effective feature selection methods to improve the clustering performance. Third, EM clustering were used in our study because of its high clustering performance and automatic determination of the number of clusters using cross validation. However, the rate of convergence of the EM clustering algorithm can be slow, and it may not be practical for very large datasets because of the computational complexity of the algorithm. Thus in future research, we could investigate some more appropriate clustering approaches and compare their performance when they are applied to our dataset. Finally, we did not focus on the differences among online participants in this study. In addition to patients, other user groups are involved in online health communities, including caregivers and health professionals. Different health-related stakeholders may consider different topics interesting. We also plan to extend our study to determine the differences in their needs and interests.
